# Supplementary material for: Graph dynamical networks for unsupervised learning of atomic scale dynamics in materials
Source: Nat Commun. 2019 Jun 17;10:2667. doi: 10.1038/s41467-019-10663-6 (PMC6573035; doi:10.1038/s41467-019-10663-6)
Supplement: Supplementary file 1 — Supplementary information [file 41467_2019_10663_MOESM1_ESM.pdf]

Supplementary Information: Graph Dynamical Networks for Unsupervised Learning of Atomic Scale Dynamics in Materials

Xie et al.

SUPPLEMENTARY NOTES

Supplementary Note 1: gated architecture in graph convolutional neural networks. The gated architecture in GCN is important for learning the local environments in a complex material system. In general, it is challenging to define bonds, i.e. the topology of the graph, in materials to capture the non-covalent interactions which may affect the atomic dynamics. We resolve this challenge by introducing the gated architecture in GCN to reweigh the strength of each connection. In the graph construction, we connect each atom with its M nearest neighbors, where the M is large enough to include non-covalent interactions. During the training, the gated architecture (or the attention layer as described in the methods section) automatically learns a weight factor that is related to the bond length and atom types. This weight factor reweighs the importance of the M nearest neighbors to center atom. As a result, we can learn a representation of the local environments in complex materials using the graphs constructed by the simple nearest neighbor approach.

Supplementary Note 2: computation of global dynamics from local dynamics in the toy system. To compute the global dynamics from local dynamics, we first assume that the transition matrix of the local Koopman model has the form,

$$\boldsymbol{K}_{\text{local}} = \begin{bmatrix} p_o & 1 - p_o \\ 1 - p_t & p_t \end{bmatrix}, \tag{1}$$

where p_o and p_t denotes the probability of the lithium atom staying in the octahedral and tetrahedral sites, respectively. Since there are 4 octahedral sites and 8 tetrahedral sites that are connected to each other in the FCC lattice, we can write the transition matrix of the

global Koopman model as,

$$\boldsymbol{K}_{\text{global}} = \begin{bmatrix} p_o & 0 & 0 & 0 & \frac{1-p_o}{8} & \frac{1-p_o}{8} & \frac{1-p_o}{8} & \frac{1-p_o}{8} & \frac{1-p_o}{8} & \frac{1-p_o}{8} & \frac{1-p_o}{8} & \frac{1-p_o}{8} \\ 0 & p_o & 0 & 0 & \frac{1-p_o}{8} & \frac{1-p_o}{8} & \frac{1-p_o}{8} & \frac{1-p_o}{8} & \frac{1-p_o}{8} & \frac{1-p_o}{8} & \frac{1-p_o}{8} & \frac{1-p_o}{8} \\ 0 & 0 & p_o & 0 & \frac{1-p_o}{8} & \frac{1-p_o}{8} & \frac{1-p_o}{8} & \frac{1-p_o}{8} & \frac{1-p_o}{8} & \frac{1-p_o}{8} & \frac{1-p_o}{8} & \frac{1-p_o}{8} \\ 0 & 0 & 0 & p_o & \frac{1-p_o}{8} & \frac{1-p_o}{8} & \frac{1-p_o}{8} & \frac{1-p_o}{8} & \frac{1-p_o}{8} & \frac{1-p_o}{8} & \frac{1-p_o}{8} & \frac{1-p_o}{8} \\ \frac{1-p_t}{4} & \frac{1-p_t}{4} & \frac{1-p_t}{4} & \frac{1-p_t}{4} & p_t & 0 & 0 & 0 & 0 & 0 & 0 & 0 \\ \frac{1-p_t}{4} & \frac{1-p_t}{4} & \frac{1-p_t}{4} & \frac{1-p_t}{4} & 0 & p_t & 0 & 0 & 0 & 0 & 0 & 0 \\ \frac{1-p_t}{4} & \frac{1-p_t}{4} & \frac{1-p_t}{4} & \frac{1-p_t}{4} & 0 & 0 & p_t & 0 & 0 & 0 & 0 & 0 \\ \frac{1-p_t}{4} & \frac{1-p_t}{4} & \frac{1-p_t}{4} & \frac{1-p_t}{4} & 0 & 0 & 0 & p_t & 0 & 0 & 0 & 0 \\ \frac{1-p_t}{4} & \frac{1-p_t}{4} & \frac{1-p_t}{4} & \frac{1-p_t}{4} & 0 & 0 & 0 & 0 & p_t & 0 & 0 & 0 \\ \frac{1-p_t}{4} & \frac{1-p_t}{4} & \frac{1-p_t}{4} & \frac{1-p_t}{4} & 0 & 0 & 0 & 0 & 0 & p_t & 0 & 0 \\ \frac{1-p_t}{4} & \frac{1-p_t}{4} & \frac{1-p_t}{4} & \frac{1-p_t}{4} & 0 & 0 & 0 & 0 & 0 & 0 & p_t & 0 \\ \frac{1-p_t}{4} & \frac{1-p_t}{4} & \frac{1-p_t}{4} & \frac{1-p_t}{4} & 0 & 0 & 0 & 0 & 0 & 0 & 0 & p_t \end{bmatrix}. \tag{2}$$

By computing the eigenvalues of $\boldsymbol{K}_{\text{global}}$, we could obtain the relaxation timescales and understand the global dynamics of the toy system. There are two major reasons for the discrepancy between the computed and observed global Koopman model: 1) the amount of MD data is not large enough to capture of full global dynamics of the lithium atom by directly learning a global dynamical model; 2) the probability of lithium atom transporting to nearby sites of the same type is not strictly zero at a given τ, so the p_o and p_t in $\boldsymbol{K}_{\text{local}}$ and the zero terms in $\boldsymbol{K}_{\text{global}}$ are approximate.

Supplementary Note 3: determination of charge for each state in PEO/LiTFSI. The charge carried by each state is determined by computing the charge integral within the first solvation shell of the Li-ions. We perform a Gaussian curve fit using the state-weighted radial distribution function of nitrogen in Supplementary Figure 3(c), since the nitrogen is the center of the TFSI anion. We assume the edge of the first solvation shell as the mean of the Gaussian curve plus 3 sigma. The charge carried by each state is computed by integrating the charge density within the first solvation using,

$$\text{Charge}_i = 1 - \int_0^{r*} g_i(r_N) \cdot 4\pi r^2 \, \mathrm{d}r + \int_0^{r*} g_i(r_{Li}) \cdot 4\pi r^2 \, \mathrm{d}r, \tag{3}$$

where r^* denotes the edge of the first solvation shell, and $g_i(r_N)$ and $g_i(r_{Li})$ denote the state-weighted radial distribution functions of nitrogen and lithium, respectively. The resulting charge for each state is summarized in Supplementary Table 1.

SUPPLEMENTARY TABLES

Supplementary Table 1. The charge carried by each state in PEO/LiTFSI.

State	0	1	2	3
Charge	+0.040	+0.262	-0.637	+0.889

Supplementary Table 2. The diffusion coefficient of each state transition in PEO/LiTFSI at $\tau = 0.8\,\mathrm{ns}$. (Unit: $10^{-7}\mathrm{cm}^2/\mathrm{s}$)

Transition	$j = 0$	$j = 1$	$j = 2$	$j = 3$
$i = 0$	3.9 ± 0.2	4.6 ± 0.1	4.6 ± 0.2	4.0 ± 0.2
$i = 1$	4.5 ± 0.2	4.3 ± 0.1	4.6 ± 0.1	4.4 ± 0.2
$i = 2$	4.4 ± 0.1	4.6 ± 0.1	3.5 ± 0.0	4.8 ± 0.7
$i = 3$	3.8 ± 0.1	3.9 ± 0.1	5.5 ± 0.6	2.8 ± 0.2

SUPPLEMENTARY FIGURES

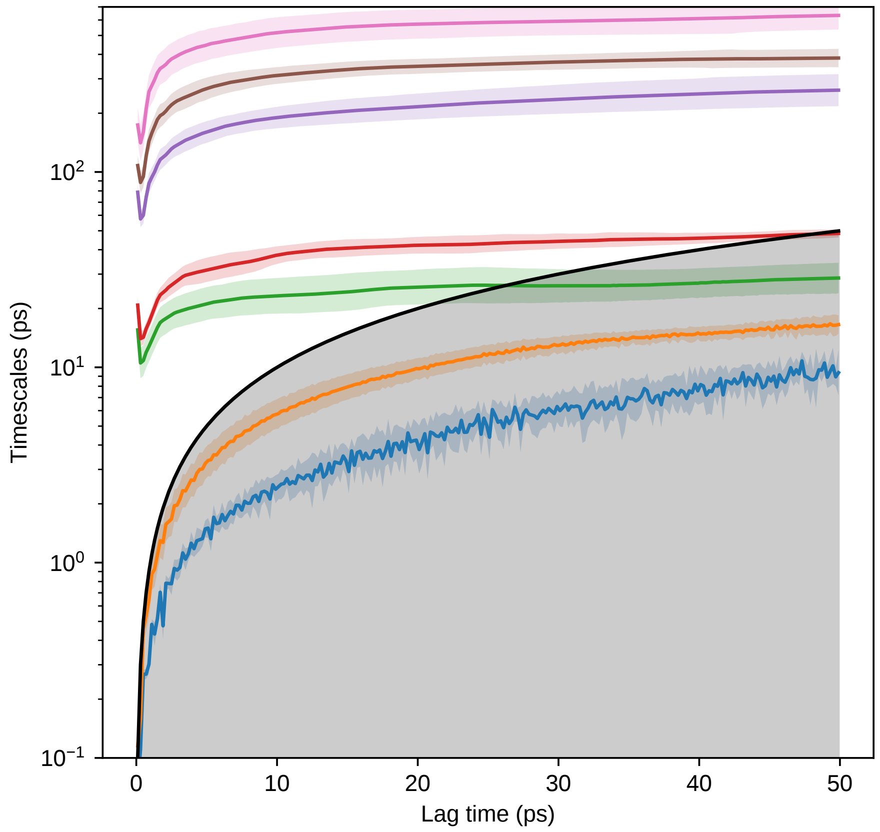

Supplementary Figure 1. Global relaxation timescales computed for lithium ion hopping in face-centered cubic (FCC) lattice with a 8 dimensional feature space.

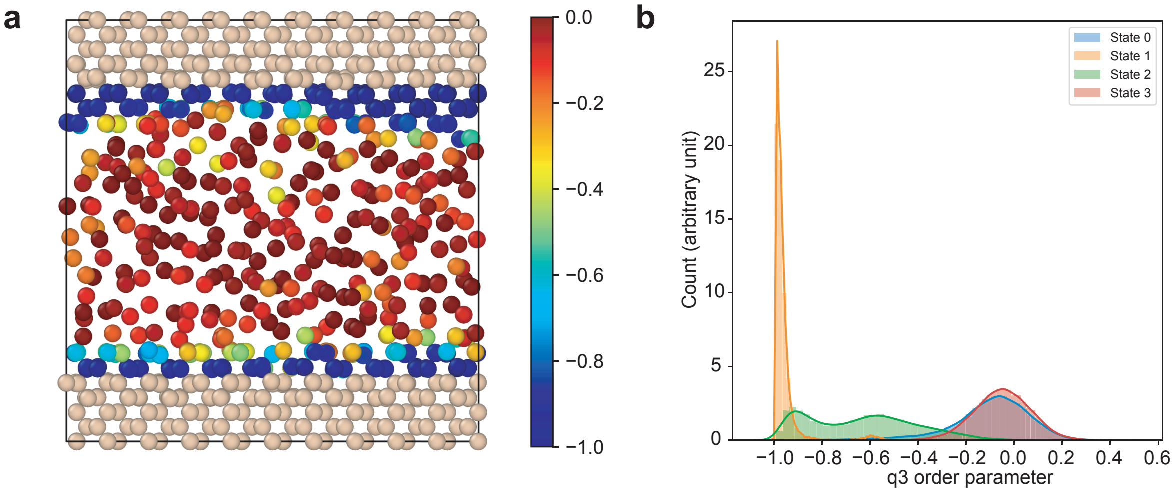

Supplementary Figure 2. Comparison between the learned states and q_3 order parameters for silicon atoms at the solid-liquid interface. (a) Cross section of the system, where the silicon atoms are color-coded with their q_3 order parameters. (b) Distribution of the q_3 order parameter for the silicon atoms of each state.

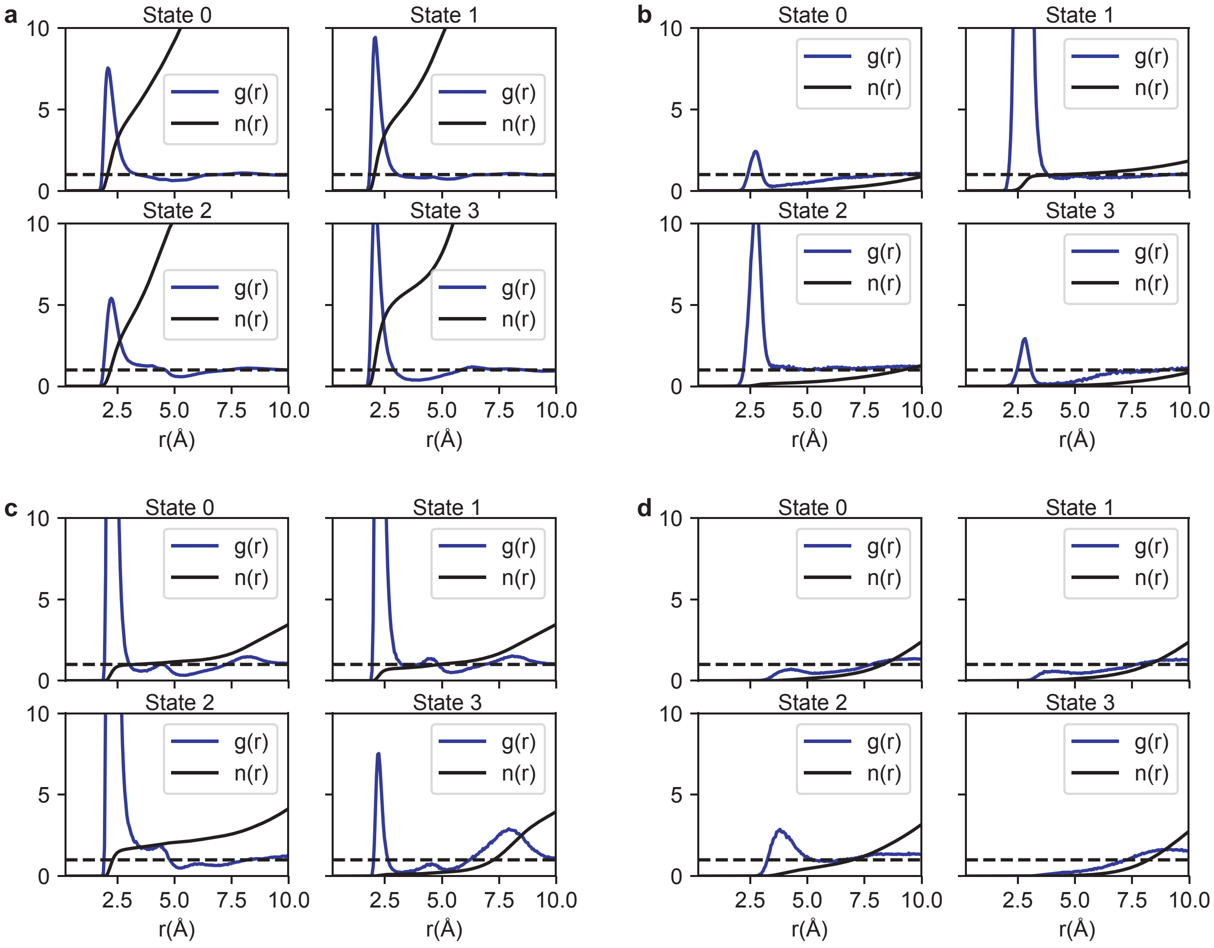

Supplementary Figure 3. State-weighted radial distribution function of Li-ions in the PEO/LiTFSI polymer electrolyte. The blue and black curves denote the radial distribution function $g(r)$ and coordination number $n(r)$ of different elements for each state, respectively. Each subfigure represents the radial distribution function of a different element: (a) oxygen, (b) hydrogen, (c) nitrogen, and (d) lithium. Note that only the hydrogen in the hydroxyl group is kept in the trajectory.
